# Adhesive Defect Monitoring of Glass Fiber Epoxy Plate Using an Impedance-Based Non-Destructive Testing Method for Multiple Structures

**DOI:** 10.3390/s17061439

**Published:** 2017-06-19

**Authors:** Wongi S. Na, Jongdae Baek

**Affiliations:** 1 Future Strategy & Convergence Research Institute, Korea Institute of Civil Engineering & Building Technology, Gyeonggi-Do 10223, Korea; 2 Highway & Transportation Research Institute, Korea Institute of Civil Engineering & Building Technology, Gyeonggi-Do 10223, Korea; jdbaek@kict.re.kr

**Keywords:** adhesive bonding, glass fiber composite, non-destructive testing, electromechanical impedance, piezoelectric transducer

## Abstract

The emergence of composite materials has revolutionized the approach to building engineering structures. With the number of applications for composites increasing every day, maintaining structural integrity is of utmost importance. For composites, adhesive bonding is usually the preferred choice over the mechanical fastening method, and monitoring for delamination is an essential factor in the field of composite materials. In this study, a non-destructive method known as the electromechanical impedance method is used with an approach of monitoring multiple areas by specifying certain frequency ranges to correspond to a certain test specimen. Experiments are conducted using various numbers of stacks created by attaching glass fiber epoxy composite plates onto one another, and two different debonding damage types are introduced to evaluate the performance of the multiple monitoring electromechanical impedance method.

## 1. Introduction

Due to their advantages such as corrosion resistance, design flexibility, durability, and high strength to weight ratio, composite materials are seeing new applications in the fields of aerospace, and civil and mechanical engineering every day. As composites offer the advantage of being able to be formed into diverse and complex shapes and sizes, they also present challenges when it comes to effectively monitoring the health of a structure. Since conventional non-destructive testing methods including acoustic emission, C-scan, and X-ray require expensive equipment and trained experts, the application of such methods to large components or structures is impractical.

For the joining of composites, adhesive bonding is usually preferable to the mechanical fastening method, particularly in the aerospace industry, in order to reduce cost and weight, and to avoid stress concentrations which can possibly result in a crack [1,2,3]. In addition, composites (e.g., glass/carbon fiber reinforced polymer) are also widely used in the field of civil engineering for the retrofitting and rehabilitation of concrete structures such as bridges and buildings. However, with the potential of debonding at the adhesive substrate interface, it is necessary to monitor the structure for any signs of defect at an early stage to prevent any possible failure. Aircrafts or civil infrastructures usually have large surface areas, which can greatly increase the cost of monitoring as a large number of sensors and measuring devices are required.

To date, one of the structural health monitoring methods known as the electromechanical impedance (EMI) method has been known to be effective for detecting damage [4,5,6,7,8,9,10,11,12,13,14,15,16,17,18,19,20,21,22,23,24,25]. It uses a single piezoelectric (PZT) transducer to actuate and sense in a high frequency range by creating a standing wave at each frequency. A PZT transducer is typically cut to a required size to carry out the EMI method, which various authors have investigated for the damage detection of composites. An advantage of the using the PZT transducer is that, owing to its small size, it can be used on almost any complex structure. Although various studies have proven that the EMI method is an effective method for detecting defects in composite structures such as delamination, most of these studies have focused on the damage detection ability, while only a few authors have investigated approaches to minimizing the cost of this monitoring method. This was achieved by using a different hardware system, which was first examined by Peairs et al. with a low-cost version of the EMI method using an FFT (fast fourier transform) analyzer [4]. Further investigations were subsequently conducted by several research groups. Mascarenas et al. [6] developed a miniaturized impedance sensor node equipped with a low-cost integrated chip that measures the impedance of the PZT transducer. Bhalla et al. [10] used a function generator with a digital multimeter which costs less than $2500 USD. Panigraphi et al. [11] used a combination of function generator and mixed signal oscilloscope to perform the EMI method with a relatively low cost. Neto et al. [13] investigated creating a portable prototype device for executing the EMI method with high stability and accuracy. A different approach of reducing the cost of the EMI method was proposed by Na and Lee [16], with a multiple piezoelectric configuration that allowed one to perform the EMI method on multiple areas using a single impedance measuring equipment.

Since most of the studies related to minimizing the cost of the EMI method have focused on developing an alternative way of measuring the impedance using various devices, this study investigates a low-cost technique for applying the EMI method to monitor several areas subjected to adhesive debonding. Up to four different composite structures were simultaneously monitored using a single frequency sweep for defects, significantly reducing the overall costs since one impedance measuring device is required per PZT transducer in general. The results obtained in this study show that this may be a promising approach for detecting adhesive debonding damage on multiple composite structures, while potentially offering the advantage of the low-cost technique to be used for any type of impedance measuring device.

## 2. Data Processing and Setup

The EMI method was first proposed by Liang et al. [26], and is a non-model based a local method that uses a piezoelectric transducer to act as both an actuator and a sensor simultaneously. The 1-D equation proposed for the EMI method is shown in Equation (1), where it proves that the electrical impedance of the PZT transducer (inverse of Y(ω)) is directly related to the mechanical impedance of the host structure ($Z_{s}\left(\omega \right)$).

(1) $$Y\left(\omega \right)=i\omega a\left(\varepsilon_{33}^{T}\left(1-i\delta \right)-\frac{Z_{s}\left(\omega \right)}{Z_{s}\left(\omega \right)+Z_{a}\left(\omega \right)}d_{3x}^{2}{\bar{Y}}_{xx}^{E}\right)$$

**Figure 1:**
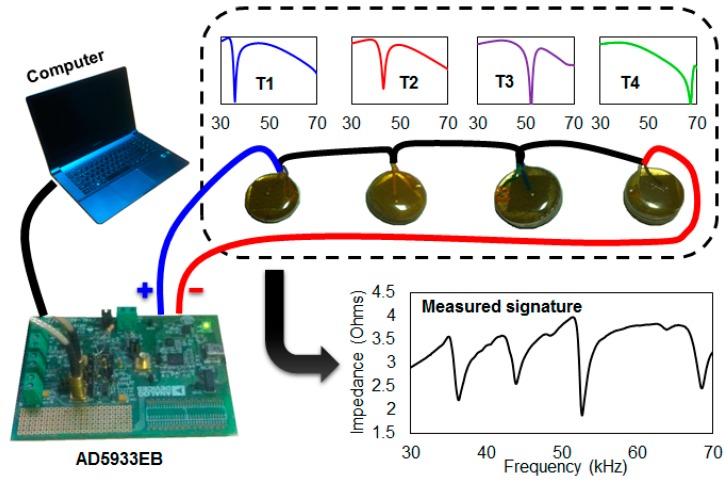
A low-cost electromechanical impedance method system setup.

In general, the EMI method uses a high frequency range under 400 kHz, which makes the method effective in outdoor environments exposed to ambient vibration noise. The high frequency range makes the EMI method less prone to vibrations, which typically happen at under 100 Hz [27]. To measure the impedance signatures, the AD5933 evaluation board manufactured by Analog Devices Co. was used throughout the study (Figure 1). The device costs less than $100 USD and has the ability to accurately measure the impedance signature up to 100 kHz with a maximum of 511 data points [28]. The EMI method is performed by connecting the AD5933 evaluation board to a computer using the USB cable provided with the package. Then, the positive and negative wires from the device are connected to the PZT transducer that is attached to the target structure, where all the experiments were conducted at a room temperature of 25 ± 0.2 °C.

The PZT transducer (model 5A4E) used in this study was purchased from Piezo Systems Inc. (www.piezo.com) and has a relative dielectric constant of 1800, a coupling coefficient of 0.35, a density of 7800 kg/m^3^, and a piezoelectric strain coefficient of −190 × 10^−12^ Vm/N. The four PZT transducers connected together in the black dotted box are explained in detail in a subsequent section.

For the damage identification process, two impedance signatures were measured before and after being damaged, and a statistical method called root-mean-square deviation (RMSD) was used to quantify the changes between the two signatures. Equation (2) shows the RMSD equation, with $Re{\left(Z_{k}\right)}_{i}$ representing the reference signature and $Re{\left(Z_{k}\right)}_{j}\;$representing the corresponding signature, where *Re* indicates that the real part of the impedance is used. Since the real part of the impedance has been reported to perform better compared to the imaginary part in terms of detecting damage [29], only the real part of the impedance was considered throughout the study.

(2) $$\mathrm{RMSD}={\left(\sum_{k=1}^{N}{\left[Re{\left(Z_{k}\right)}_{j}-Re{\left(Z_{k}\right)}_{i}\right]}^{2}/\sum_{k=1}^{N}{\left[Re{\left(Z_{k}\right)}_{i}\right]}^{2}\right)}^{1/2}$$

## 3. Limitations of the Conventional PZT Attachment EMI Method

### 3.1. Damage Detection against Debonding Damage

**Figure 2:**
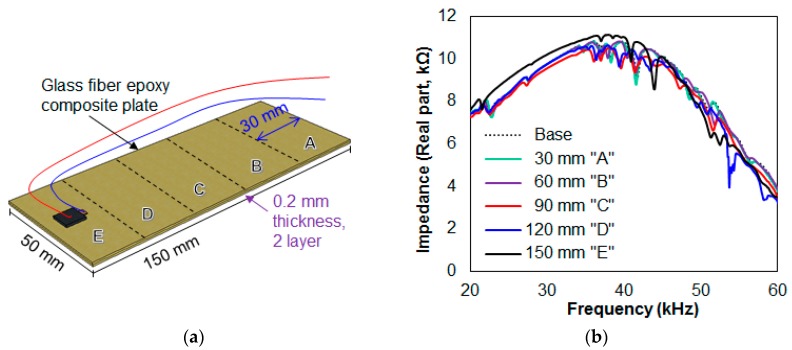
(**a**) First debonding test scenario; (**b**) results from the first debonding test.

**Figure 3:**
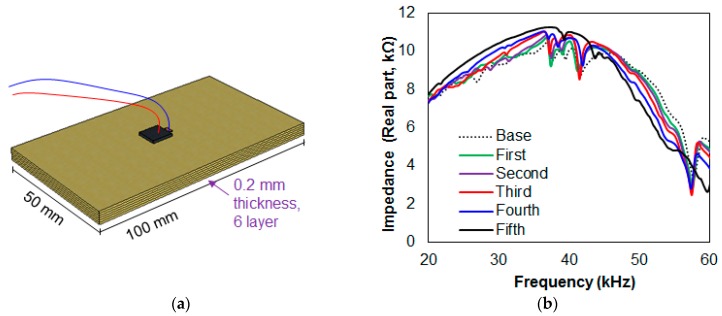
(**a**) Second debonding test scenario; (**b**) results from the second debonding test.

In this subsection, two different scenarios were created for detecting debonding damage of composite plates attached together using a commercial epoxy adhesive (Loctite Quick Set). The glass fiber epoxy composite plates used throughout the study were purchased from a domestic company, ArtRyx (www.artryx.com), and had a thickness of 0.2 mm. Figure 2a shows the first test scenario consisting of two 50 mm × 150 mm composites attached together using the epoxy adhesive with a 15 mm square PZT transducer attached on top of the plate. The test was to artificially create 30 mm of debonding at each step using a chisel (labeled “A” to “E”) starting from the opposite side of the PZT (labeled “A”) where an impedance signature was measured after every 30 mm of debonding until the bottom plate was completely detached (labeled “E”). The impedance signatures are shown in Figure 2b in the frequency range of 20 kHz to 60 kHz, where this range was manually selected by observation. The signature in the 20 kHz to 40 kHz range shifts in the upward direction at 150 mm debonding (black line) where the attached PZT transducer lies above this region. This shows that the impedance signature at a certain frequency range shifts in the vertical direction when debonding occurs directly below the PZT transducer. This behavior can be verified with the next test scenario in Figure 3. Thus, it becomes possible for one to identify debonding in the thickness direction using this feature as one of the damage identification steps.

Calculating the RMSD values for the damaged impedance signatures compared to the reference signature proves that the damage intensity was successfully identified, with the values being 1.37%, 2.48%, 4.37%, 5.71%, and 7.08% with progressive damage. Fitting these values with a straight line results in an R^2^ value of 0.995, showing a virtually perfect relationship between the damage intensity (debonding length for this case) and the RMSD values.

Figure 3a shows the second test scenario, in which six composites of size 50 mm × 100 mm were stacked together using the epoxy adhesive. Then, a 15-mm square PZT transducer was attached to the top of the stack using the same adhesive where the stack was left for 48 h to ensure full curing. This test scenario was to detach composite plates one at a time for the first five plates with the impedance signatures being measured at each step, finally leaving a single plate with the PZT attached. Figure 3b shows the impedance signatures obtained from the above test where the signatures were measured from 20 kHz to 60 kHz in the 100 Hz step. By observation, the peaks shift in either the left or right direction with amplitudes changing in general. In addition, when compared with the reference signature (represented by the black dotted line) the whole impedance signature tends to shift in the upward direction up to about 45 kHz, then the signature shifts in the downward direction in the 45 kHz to 60 kHz range.

Calculating the RMSD values results in 4.32%, 5.38%, 7.83%, 10.09%, and 14.38% for debonding the first five composite plates. The intact case was used as the reference signature, and all the other signatures were compared with this reference signature in the frequency range from 20 kHz to 60 kHz. When a linear line of best fit was applied using these five RMSD values, the coefficient of determination (R^2^) was 0.953, showing a very strong relationship between the damage intensity (number of detached plates for this case) and the RMSD values.

From the two tests, damage was successfully identified as expected using the EMI method subjected to the two different ways of artificial debonding damage. However, only one area can be monitored through the conventional way of performing the EMI method. From the results, it can be seen that the resonance peaks and the impedance signature itself change throughout the frequency range from 20 kHz to 60 kHz. If one were to divide the above frequency range into multiple regions where the impedance signature in that region reacts to a specific area, it becomes possible for one to monitor multiple areas with a single frequency sweep. Thus, in order to apply this approach in conjunction with the EMI method, its details are explained in the following section.

### 3.2. Monitoring up to Four Different Areas

Na et al. [9] proposed a method of monitoring up to three metal plates subjected to notch damage using PZT transducers of different sizes. The main idea was that the differently-sized PZT transducers had different resonance frequency ranges, and connecting them together with the steel wires made multiple area monitoring possible. Since any damage to a target structure changes its vibration behavior, this affects the attached steel wire, resulting in the change in the impedance signature of the PZT that is secured at the other end of the steel wire [9]. However, different resonance frequencies were achieved by utilizing different PZT sizes of 20 mm, 25 mm, and 35 mm with a width of 10 mm, which can limit the number of areas that can be monitored.

In this study, multiple monitoring was achieved by connecting several PZT transducers of the same size in conjunction with differently-sized metal objects to create resonances in different ranges. In a previous figure (Figure 1), this can be seen inside the black dotted line box where the four PZT transducers are connected in series with metal discs of different thicknesses attached to each of the transducers. The four PZT transducers are labelled T1, T2, T3 and T4 for the study, where the thicknesses of the metal objects were 2 mm, 3 mm, 5 mm, and 7 mm, respectively. In addition, the four impedance signatures shown inside the dotted box shows the transducers individually measured where different resonance frequency ranges were observed for all transducers. The difference in the resonance frequency ranges was due to the different metal thicknesses where using a thicker metal disc results in higher resonance frequency ranges. Directly below the dotted box in the figure shows the measured impedance signatures of T1 to T4 connected in a series for the setup where four resonance peaks are observed with a single frequency sweep. Thus, one can simultaneously monitor up to four areas using this setup, as damaging an area attached to a specific transducer will affect its impedance signature (T1, T2, T3, or T4).

## 4. Experimental Setup

### 4.1. Progressive Debonding in X-axis Direction of Three Composites

**Figure 4:**
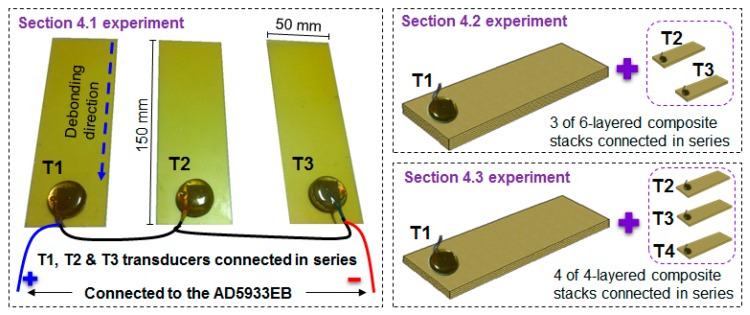
Test specimen setup for Section 4.1, Section 4.2 and Section 4.3.

Using the T1, T2, and T3 transducers connected in series, each of them was attached to a two-layered glass fiber epoxy composite plate with a size of 150 mm × 50 mm × 0.2 mm, as shown in Figure 4. The experiment for this subsection involved debonding the bottom composite plate in 30 mm increments, until 150 mm of debonding was achieved, identical to the test method carried out for Figure 2a. The impedance signature was measured after each step, resulting in six signatures including the reference signature (intact case). This process was conducted for the setup shown by damaging the composite stack with the T1 attached only, and then this process was repeated by damaging the stack with T2 attached only, then T3. From this point onwards, these three test scenarios will be referred to as Case 1, Case 2, and Case 3, respectively.

### 4.2. Debonding in the Thickness Direction of Three Composites

With the three transducers used in the previous Section 4.1, another experiment was conducted by creating an artificial debonding in the thickness direction (identical test method to Figure 3a). Here, six composite plates with a size of 100 mm × 50 mm × 0.2 mm were attached to one another using the epoxy adhesive to create a six-layered composite stack. Then, two more identical stacks were made to be connected in series. The experiment consisted of detaching a composite plate from a stack one at a time for the first five plates while measuring the impedance signatures, resulting in six signatures in total including the reference signature. The experiment was conducted by damaging a single stack only in the order of T1, T2, and T3 attached composites, and these are defined as Case 4, Case 5, and Case 6, respectively, for the study.

### 4.3. Debonding Damage Combinations in the Thickness Direction of Four Composites

The two previous Section 4.1 and Section 4.2 investigated damaging a single stack while the other two stacks remain undamaged. To further evaluate the performance of the transducers connected in series that were subjected to debonding, four transducers (T1, T2, T3 and T4) were connected in series for the experiment in this section. Instead of creating debonding damage onto a single stack (a 4-layered composite), two stack damage combinations were selected for the analysis. With four composite plates, six different damage combinations (^4^C_2_) were created to carry out the EMI method, which is defined as Case 7. Afterwards, three stack combinations were selected and damaged with four different damage combinations (^4^C_3_), defined as Case 8 for the study.

## 5. Results and Discussions

### 5.1. X-axis Direction Debonding Results for Three Composites

**Figure 5:**
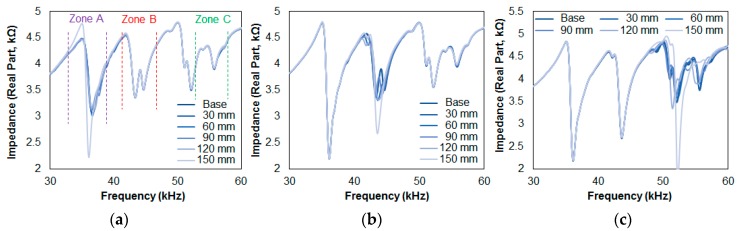
Impedance signature results for: (**a**) Case 1; (**b**) Case 2; (**c**) Case 3.

Figure 5 shows the impedance signature results for Cases 1, 2, and 3 in the resonance frequency range from 30 kHz to 60 kHz. At first glance, the impedance signatures seem to change depending on the specific stack that is damaged. For example, a significant change in the impedance signature is observed at 37 kHz in Figure 5a as the damage was introduced to the T1 attached composite plate only. So, in defining which one of the composite plates is being damaged, ideally a frequency range should be manually selected for the user to identify where the damage has occurred. Thus, for this section, three different frequency ranges were manually chosen by seeking for ranges containing multiple peaks and defined as “Zone A,” “Zone B,” and “Zone C,” representing resonances for the three transducers. These are 32 kHz to 39 kHz, 41 kHz to 46 kHz, and 52 kHz to 58 kHz for Zones A, B and C, respectively, as shown in Figure 5a. With a single sweep, three RMSD values were calculated to identify which one of the plates had been damaged.

In Figure 5a, representing Case 1, the impedance signatures in the frequency range at around 37 kHz changes due to damage. However, the variations in the signatures are very small at 120 mm debonding, while a significant increase in the peak is observed at 150 mm debonding. In Figure 5b, which represents the results from Case 2, a significant change in the impedance signatures is observed for the resonance located at 42 kHz, where the two peaks (clearly shown in Figure 5a in Zone B) have changed into a single peak. In Figure 5c for Case 3, the impedance signatures change more significantly compared to the previous two figures in Zone C. One of the reasons for this is that the resonance in this range has more peaks, causing the signatures to change more severely compared to a signature with fewer resonance peaks.

Table 1 shows the RMSD values for Figure 5, where all the impedance signatures were compared to a reference signature (intact case). With Case 1, Zone A results in an RMSD value starting from 0.56% with the first 30 mm of debonding and ends at 10.23% after 150 mm of debonding. The values 0.51%, 1.73%, and 1.58%, which correspond to 60, 90, and 120 mm of debonding, respectively, show that the relationship between the increase in the damage intensity (debonding length) and the RMSD values is weak (R^2^ of 0.612). Nevertheless, the EMI method has successfully identified the debonding damage for Case 1 with the RMSD values for Zone B and Zone C all being lower than 1%. Thus, if one were to define a threshold value of 1%, the T1 transducer would be able to detect the debonding damage with a sensing radius of approximately 90 mm for this setup (since the RMSD values failed to detect up to 60 mm of debonding with the values of 0.56% and 0.51%). For Case 2, the RMSD values for Zone A and C are all below 1%, with the highest value being 0.75%. The first 30 mm of debonding damage results in 0.49% (Zone B), and ends at 8.81% with 150 mm of debonding damage. In addition, the RMSD values of 4.54%, 3.15%, and 5.41% for 60 mm, 90 mm, and 120 mm of debonding damage, respectively, show a better relationship between the damage intensity and the RMSD values for this case (R^2^ of 0.821). For Case 3, the RMSD values are less than 1% for the two stacks without any damage (Zone A and B). The RMSD values for Zone C start at 0.94% and increase with the debonding length, ending at a value of 14.16% with the middle values being 1.79%, 2.77%, and 5.13%, showing a good relationship compared to damage intensity (R^2^ of 0.767).

Although the debonding damage was successfully identified using this configuration, the relationship between the RMSD values and damage intensity seemed to be sacrificed at the cost of multiple monitoring (since the R^2^ values from Section 3 were 0.995 and 0.953 using the conventional method of attaching the PZT transducer onto a host structure). One reason for this is due to the fact that there were insufficient resonance peaks in the impedance signature, as Cases 2 and 3 showed a better relationship (higher R^2^ values) compared to Case 1 due to the existence of additional peaks. This suggests that the number of peaks is a very important factor when the EMI method is used.

### 5.2. Thickness Direction Debonding Results for Three Composites

**Figure 6:**
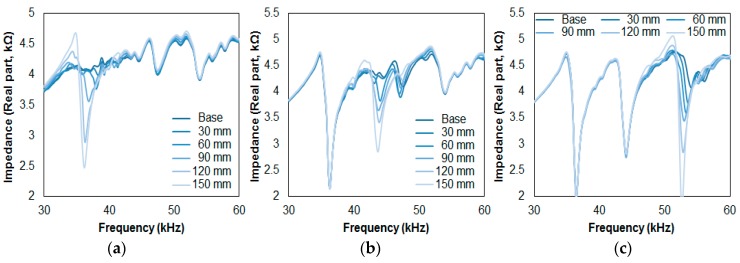
Impedance signature results for: (**a**) Case 4; (**b**) Case 5; (**c**) Case 6.

Figure 6 shows the impedance signatures from Cases 4, 5, and 6, where the impedance signatures in each zone change depending on which composite stack has been damaged. For Figure 6a, representing Case 4, the change is clearly observed in Zone A with virtually no changes in Zones B and C. Compared to Figure 5a, the variations in the impedance signature are greater, proving once again that the debonding in the thickness direction shows better performance. Similar behavior is shown for Cases 5 and 6 where the impedance signatures change according to their corresponding zone, Zone B for Figure 6b and Zone C for Figure 6c.

Table 2 shows the calculated RMSD values at each zone for Figure 6. For Case 4, the RMSD values for Zone A show an increase, starting from 1.24% with the first plate detachment and ending at a value of 15.89% with the last plate detachment. For Zones B and C, the highest RMSD values go up to 1.91% and 1.51%, respectively. Thus, by setting a threshold value of 2% for this section, a damaged stack can be differentiated from an undamaged stack. However, since the first detachment of the composite plate for Zone A in Case 4 resulted in an RMSD value of 1.24%, the threshold value ignores this result, failing to identify the first debonding damage for this case. Nevertheless, the EMI method successfully detected the debonding damage for the remaining detachments. For Case 5, the RMSD values for the undamaged composite stack (Zones A and C) are all less than 1.80%. For Zone B, the RMSD values start at 1.70% and end at 15.95%; again, failing to identify damage for the first plate debonding (less than the threshold value of 2%). For Case 6, the undamaged stacks (Zones A and B) result in the RMSD values all being less than 1.15%, while the RMSD values for Zone C start at 4.47% and end at 23.06%, perfectly distinguishing all damaged stacks from the undamaged stacks.

Overall, the difference in the metal layer thickness for T1 (2 mm), T2 (3 mm), and T3 (5 mm) can cause the highest RMSD values for each case to differ due to their different properties. However, the RMSD values for Zone A in Case 4 and Zone B in Case 5 show similarity in values (1.24% with 1.70%, 3.25% with 4.58%, 5.23% with 7.74%, 10.81% with 10.26%, and 15.89% with 15.95%), but the values for Zone C in Case 6 show a large difference (4.47%, 7.86%, 10.73%, 15.83%, and 23.06%), experimentally showing that another factor is involved which causes such an outcome. In general, the resonance frequency range selection is selected manually after attaching a PZT transducer onto a host structure. This is due to the fact that the resonance frequency range is unknown, and must be found manually. In addition, the number of peaks and its amplitudes are usually random with the PZT attachment. Re-attaching the same PZT transducer onto the same host structure can cause the impedance signature shape to completely change due to its high sensitivity, and this is what makes the EMI method extremely complex in terms of pinpointing the damage location, type, and size.

### 5.3. Results for Debonding Damage Combinations for Four Composites

**Figure 7:**
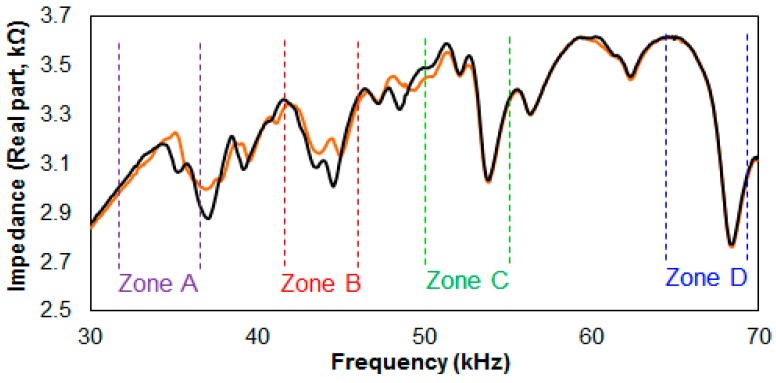
Impedance signatures from debonding T1 and T2 transducer attached stacks.

Figure 7 shows the Case 7 impedance signature results acquired from the first debonding combination of damaging the stacks with T1 and T2 transducers attached. One may notice that the impedance signature amplitudes have been reduced with the extra attachment of the PZT transducer when compared to a previous figure. This is due to the fact that same amount of energy is divided across the series circuit, and adding another PZT transducer will reduce the amplitudes in general, as shown in this study. With the additional T4 transducer introduced to the series circuit, the frequency ranges for Zones A, B and C were re-defined with Zone D for the RMSD calculations. These were 32 kHz to 37 kHz, 42 kHz to 46 kHz, 50 kHz to 56 kHz, and 65 kHz to 70 kHz for Zones A, B, C, and D, respectively, as shown in the figure. Through observing the change in the impedance signature inside its zone area, it can be confirmed which composite stack has been damaged. The impedance signature change in Figure 7 is very clear for Zone A and Zone B, indicating that the T1 and T2 transducer attached stacks have been damaged, while Zone C experiences a small change (which can be ignored by setting a threshold value) and Zone D has virtually no variation.

**Figure 8:**
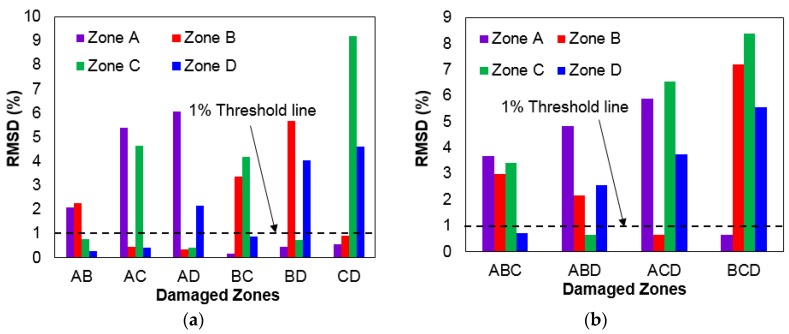
Bar graphs of RMSD values from impedance signatures from: (**a**) Case 7; (**b**) Case 8.

After acquiring all the impedance signatures from testing Case 7, the RMSD values were calculated to plot a bar graph, as shown in Figure 8a. The four different zones are represented by the different colors shown in the figure with the X-axis labels (“AB” to “CD”) showing which zones are damaged. The results are very positive, as the RMSD values at each zone successfully indicate which stacks are damaged. For example, “AB” shows Zone A and Zone B with over 2% RMSD with the remaining Zone C and Zone D resulting in the RMSD values of less than 1%. Thus, setting a threshold value of 1% and neglecting any values under this point can differentiate a damaged stack from an undamaged stack. Another observation can be discussed here with the maximum RMSD values calculated for each zone. The highest RMSD values calculated for Zones A, B and C were 6.05%, 5.66%, and 9.2%, but the highest RMSD value for Zone D was 4.59%, experimentally proving that a thicker metallic layer does not always result in a higher RMSD value.

The bar graph shown in Figure 8b was plotted using the RMSD values acquired from Case 8, where three stacks of the four stacks connected in series are damaged. Again, setting the threshold value of 1% allows one to easily identify which stacks have been damaged when all the damaged stacks have RMSD values of over 2%. The highest RMSD values obtained for Zones A, B, C, and D were 5.89%, 7.21%, 8.39%, and 5.55%, which experimentally proves once more that a thicker metallic layer does not always perform better. Another observation made here that the RMSD values are much higher in general for “BCD” compared to “ABC”. This is due to the fact that the experiment starts with all four stacks connected in series, with each stack having four layers of composites. The layers decrease as the experiment is conducted, causing the amplitude of the resonance to increase, as shown in Figure 6. Thus, with a larger amplitude, a more severe change in the impedance signatures will occur, causing the calculated RMSD values to be higher.

## 6. Conclusions

In this study, multiple monitoring using metallic discs with different thicknesses was used to monitor up to four separate glass epoxy composites subjected to debonding damage. The limitation of the conventional approach of attaching a single PZT transducer onto a host structure was realized in the first part of the study, where only a single area could be monitored with the use of the AD5933 evaluation board. Two different types of debonding damage were introduced, where the first case involved creating an artificial debonding in the X-axis direction, and the second case involved debonding in the thickness direction. From the results, it was experimentally proven that the EMI method detected the debonding in the thickness direction better, with the impedance signatures showing significant changes subjected to the damage.

The second part of the study was to apply the multiple monitoring approach by using three PZT transducers that were individually attached to a metal disc with varying thicknesses to achieve various resonance frequency ranges. Each of the transducers was attached to each of the three composite stacks that were attached together using a commercial epoxy adhesive. The test was to create two different types of debonding identical to the first part of the study, where the positive results revealed the possibility of monitoring multiple areas with a single impedance signature sweep. The frequency range from 30 kHz to 60 kHz was used for this section, and this was divided into three different areas as Zone A (32–39 kHz), Zone B (41–46 kHz), and Zone C (52–58 kHz) to represent each of the three composite stacks.

The last part of the study involved introducing an additional PZT transducer with a thicker metallic layer to monitor four composite stacks. Two different cases were tested in this part; the first case introduced debonding onto two stacks, resulting in six different combinations (^4^C_2_); and the second case created debonding onto three stacks, resulting in four different combinations (^4^C_3_). By re-defining the frequency range for Zone A to Zone D, the EMI method successfully identified which stacks had been damaged by setting a threshold value of 2%. Thus, by using the multiple monitoring approach presented in this study, one is able to monitor up to four different areas for detecting the debonding of composite structures.

## Figures and Tables

**Table 1:** Calculated RMSD values for Cases 1, 2, and 3.

		30 mm	60 mm	90 mm	120 mm	150 mm
**Case 1**	Zone A	0.56	0.51	1.73	1.58	10.23
	Zone B	0.11	0.18	0.17	0.44	0.75
	Zone C	0.16	0.21	0.30	0.41	0.59
**Case 2**	Zone A	0.19	0.25	0.24	0.38	0.52
	Zone B	0.49	4.54	3.15	5.41	8.81
	Zone C	0.11	0.17	0.35	0.51	0.75
**Case 3**	Zone A	0.34	0.36	0.42	0.42	0.47
	Zone B	0.60	0.69	0.72	0.70	0.96
	Zone C	0.94	1.79	2.77	5.13	14.16

**Table 2:** Calculated RMSD values for Cases 4, 5, and 6.

		First	Second	Third	Fourth	Fifth
**Case 4**	Zone A	1.24	3.25	5.23	10.81	15.89
	Zone B	0.75	1.02	0.89	1.37	1.91
	Zone C	0.28	0.51	0.73	0.93	1.51
**Case 5**	Zone A	0.36	0.72	1.04	1.37	1.80
	Zone B	1.70	4.58	7.74	10.26	15.95
	Zone C	0.52	0.77	0.72	1.08	1.36
**Case 6**	Zone A	0.12	0.23	0.26	0.30	0.49
	Zone B	0.21	0.53	0.54	0.66	1.15
	Zone C	4.47	7.86	10.73	15.83	23.06
